# Evaluation of the PREDIGT score’s performance in identifying newly diagnosed Parkinson’s patients without motor examination

**DOI:** 10.1038/s41531-022-00360-5

**Published:** 2022-07-29

**Authors:** Juan Li, Tiago A. Mestre, Brit Mollenhauer, Mark Frasier, Julianna J. Tomlinson, Claudia Trenkwalder, Tim Ramsay, Douglas Manuel, Michael G. Schlossmacher

**Affiliations:** 1 Neuroscience Program, Ottawa Hospital Research Institute, Ottawa, ON Canada; 2 Clinical Epidemiology Program, Ottawa Hospital Research Institute, Ottawa, ON Canada; 3 University of Ottawa Brain and Mind Research Institute, Ottawa, ON Canada; 4 Division of Neurology, Department of Medicine, The Ottawa Hospital, Ottawa, ON Canada; 5 Department of Medicine, Faculty of Medicine, University of Ottawa, Ottawa, ON Canada; 6 Elena-Paracelsus Klinik, University of Goettingen, Kassel, Germany; 7 Michael J. Fox Foundation for Parkinson’s Research, New York, USA; 8 Department of Cellular and Molecular Medicine, University of Ottawa, Ottawa, ON Canada; 9 School of Epidemiology and Public Health, Faculty of Medicine, University of Ottawa, Ottawa, ON Canada; 10 Methods Centre, Ottawa Hospital Research Institute, Ottawa, ON Canada; 11 Department of Family Medicine, Faculty of Medicine, University of Ottawa, Ottawa, ON Canada

**Keywords:** Parkinson's disease, Risk factors, Epidemiology, Diagnostic markers

## Abstract

Several recent publications described algorithms to identify subjects with Parkinson’s disease (PD). In creating the “PREDIGT Score”, we previously developed a hypothesis-driven, simple-to-use formula to potentially calculate the incidence of PD. Here, we tested its performance in the ‘De Novo Parkinson Study’ (DeNoPa) and ‘Parkinson’s Progression Marker Initiative’ (PPMI); the latter included participants from the ‘FOllow Up persons with Neurologic Disease’ (FOUND) cohort. Baseline data from 563 newly diagnosed PD patients and 306 healthy control subjects were evaluated. Based on 13 variables, the original PREDIGT Score identified recently diagnosed PD patients in the DeNoPa, PPMI + FOUND and the pooled cohorts with area-under-the-curve (AUC) values of 0.88 (95% CI 0.83–0.92), 0.79 (95% CI 0.72–0.85), and 0.84 (95% CI 0.8–0.88), respectively. A simplified version (8 variables) generated AUC values of 0.92 (95% CI 0.89–0.95), 0.84 (95% CI 0.81–0.87), and 0.87 (0.84–0.89) in the DeNoPa, PPMI, and the pooled cohorts, respectively. In a two-step, screening-type approach, self-reported answers to a questionnaire (step 1) distinguished PD patients from controls with an AUC of 0.81 (95% CI 0.75–0.86). Adding a single, objective test (Step 2) further improved classification. Among seven biological markers explored, hyposmia was the most informative. The composite AUC value measured 0.9 (95% CI 0.88–0.91) in DeNoPa and 0.89 (95% CI 0.84–0.94) in PPMI. These results reveal a robust performance of the original PREDIGT Score to distinguish newly diagnosed PD patients from controls in two established cohorts. We also demonstrate the formula’s potential applicability to enriching for PD subjects in a population screening-type approach.

## Introduction

Parkinson’s disease (PD) remains an incurable neurodegenerative condition. The worldwide prevalence of PD is estimated at 6.1 million^1^ and its incidence is expected to double by 2030^2^. As of 2022, no disease-modifying agents have been approved and no preventive therapy exists to delay the onset of clinical PD in those at higher risk. It is widely assumed that an earlier diagnosis of PD, including in its prodromal phase, would enable future breakthroughs in disease modification^3^. To this end, establishing a reliable working diagnosis early in its course appears essential.

Predicting the future incidence of PD in neurologically healthy individuals is challenging due to the incomplete understanding of the disease’s aetiology and the limited availability of large, prospective cohorts of neurologically healthy individuals followed for incident PD. Recently, a small number of models have been developed using data analyses-driven approaches to identify subjects in the earliest stages of PD^4–12^. Berg et al. presented a three-step approach to determine the risk of incident PD^4^. The approach by Nalls et al.^5^ distinguished PD patients from healthy controls (HC) in the ‘Parkinson’s Progression Marker Initiative’ (PPMI) cohort with an area-under-the-curve (AUC) value of 0.92. External validation of this model showed good classification of PD (AUC ≥0.9). In the Schrag et al. approach^6^, neurological and psychiatric variables, such as tremor, rigidity, cognitive decline and depression, were integrated together with autonomic variables. This model was recently refined to enhance the prediction algorithm’s performance^7^.

On behalf of the International Movement Disorder Society (MDS), Berg et al. proposed an algorithm to diagnose subjects in a prodromal stage of PD^8,9^. The MDS Research Criteria have since been validated in three prospective studies: an elderly community-based cohort^10^, a longitudinal cohort of REM sleep behavior disorder (RBD) subjects^11^, and a genetically characterized cohort of mutant *LRRK2* allele carriers^12^.

In parallel, we had designed the PREDIGT Score model as a hypothesis-driven approach to quantify disease incidence based on established risk factors linked to the pathogenesis of PD^13^. In this approach, the selection of variables and their coefficients was not restricted to any particular dataset (or a specific cohort). Instead, the PREDIGT model was based on: (1) the hypothesis that PD is a complex disorder requiring interactions between several risk elements to promote disease; (2) an umbrella review of 75 meta-analyses to estimate the effect size of each identified variable; and (3) a model for pathogenesis that sees the gradual evolution from a healthy state to a prodromal phase on to the manifestation of cardinal signs, which subsequently lead to the diagnosis of PD^14^.

We had postulated that the development of ‘idiopathic’ PD could be explained by interacting contributions from five risk categories: Exposome, as the sum of environmental exposures (E); genetic susceptibility (DNA variants; D); the presence of tissue changes from documented gene-environment Interactions (I); sex/Gender (G); and age, as in the passage of Time (T). We had further proposed -and mathematically modeled- that a final risk score could be calculated by assigning values to each category, as computed by the simple formula of: P_R_ = (E + D + I) x G x T. For select risk elements, where details of variables remain unknown, such as for a person’s exposome history (E) within the nasal cavity and gastrointestinal tract, or for the specifics of inherited genetic variants (D), we had identified simple-to-impute surrogates, such as hyposmia, constipation, and family history, respectively^13^. Motor assessment-based data from validated questionnaires and neurological examinations, such as captured by the UPDRS subscales, were not included in the original model. The reason for this decision -when creating the model- had been the related goal to calculate future PD incidence in neurologically still healthy individuals; this had also been the stated goal of the MDS Research Criteria for Prodromal Parkinson’s Disease effort^8,9^.

Here, as a first step to evaluate the PREDIGT Score’s performance, we set out to assess its discriminative ability to identify persons that have been diagnosed at an early stage of PD *versus* HC subjects. Further, we also designed and tested a practical plan to apply the PREDIGT Score to potential population-type screening: In Step 1, we used self-reported answers to a standardized ‘PREDIGT Questionnaire’ for the enrichment of individuals with a higher risk profile, followed in Step 2 by the addition of one objective, clinical measurement from a group of seven biological markers.

## Results

In pursuit of these two objectives, we used data from two well-established, previously characterized case-control studies, *i.e*., the ‘De Novo Parkinson Study’ (DeNoPa)^15^ and PPMI^16^. Data from the ‘FOllow Up Persons with Neurologic Disease Study’ (FOUND)^17^, a follow-up study for a subset of PPMI participants, were used to supplement the environmental exposure variables that had not been collected during the initial enrollment phase of the PPMI study. The mean stage of disease severity in PD patients was documented by the Hoehn and Yahr score at the time of study enrollment (Table 1). Of note, no subjects in the DeNoPa cohort were enrolled in the PPMI (FOUND) study and vice versa.

**Table 1:** Demographic characteristics of adults enrolled in the single-centre DeNoPa and multi-centric PPMI (and FOUND) cohorts.

	DeNoPa			PPMI^a^			FOUND^a^		
	Case	Control	*p*	Case	Control	*p*	Case	Control	*p*
No. of participants	135 (55.1)	110 (44.9)		428 (68.59)	196 (31.41)		141 (65.28)	75 (34.72)	
Male	88 (65.19)	67 (60.91)	0.49	280 (65.42)	126 (64.29)	0.78	94 (66.67)	51 (68)	0.84
Age at baseline, years	66 (40–84)	65.5 (44–84)	0.85	63 (34–85)	62.5 (31–84)	0.52	61 (36–82)	60 (31–80)	0.74
Male	66 (40–82)	67 (44–84)	0.69	63 (35–85)	63 (31–83)	0.69	63 (36–79)	61 (31–80)	0.64
Female	66 (41–84)	64 (51–74)	0.51	61 (34–82)	60 (31–84)	0.61	56 (39–82)	56 (32–71)	0.74
10-year age brackets, No. (%)									
<40	0 (0)	0 (0)		12 (2.8)	8 (4.08)		3 (2.13)	3 (4)	
40–50	8 (5.93)	2 (1.82)		37 (8.64)	21 (10.7)		17 (12.1)	8 (10.7)	
50–60	35 (25.9)	22 (20)		113 (26.4)	54 (27.6)		45 (31.9)	26 (34.7)	
60–70	45 (33.3)	57 (51.8)		171 (40)	68 (34.7)		54 (38.3)	23 (30.7)	
70–80	41 (30.4)	28 (25.4)		87 (20.3)	38 (19.4)		21 (14.9)	14 (18.7)	
80+	6 (4.44)	1 (0.91)		8 (1.87)	7 (3.57)		1 (0.71)	1 (1.33)	
**Hoehn and Yahr Stage**	**UPDRS**			**MDS UPDRS**			**MDS UPDRS**		
0	0 (0)	110 (100)		0 (0)	193 (98.5)		0 (0)	75 (100)	
1	33 (24.4)			189 (44.2)	2 (1.02)		68 (48.2)		
1.5	40 (29.6)			n.a.			n.a.		
2	25 (18.5)			237 (55.4)			72 (51.1)		
2.5	26 (19.3)			n.a.			n.a.		
3	11 (8.15)			2 (0.467)			1 (0.709)		

^a^For PPMI and FOUND data, only participants classified as “Parkinson’s disease” and “Healthy Control” were included.

Data are No. (%), or median (range). The reported *p*-values represent the significance from corresponding Kruskal–Wallis Tests.

DeNoPa = *De Novo* Parkinson Study. PPMI = Parkinson’s Progression Marker Initiative. FOUND = Follow Up Persons with Neurologic Disease Study. MDS = International Parkinson and Movement Disorder Society. UPDRS = Unified Parkinson’s Disease Rating Scale. n.a. not available.

**Fig. 1:**
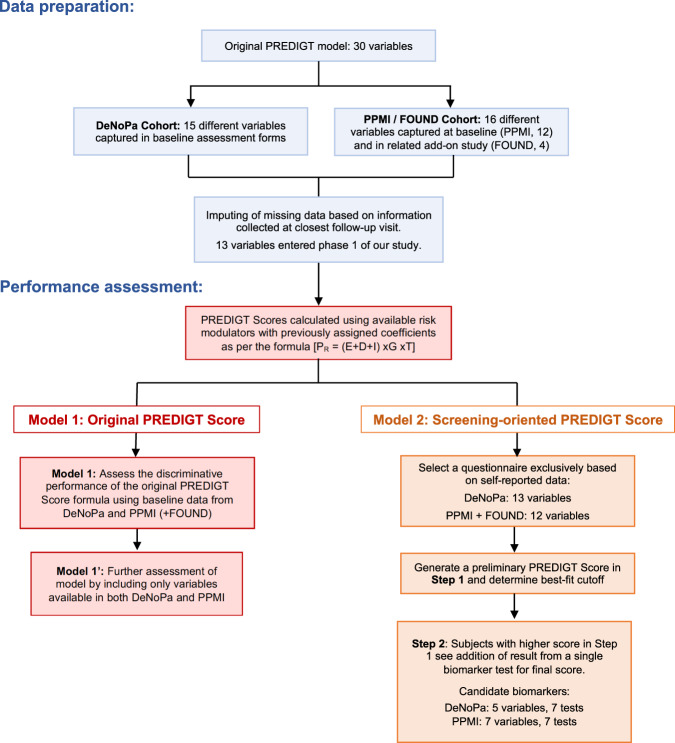
Workflow of this study. DeNoPa denotes De Novo Parkinson Study; PPMI Parkinson’s Progression Marker Initiative, FOUND FOllow Up persons with Neurologic Disease cohort, AUC area under the receiver operating characteristic curve.

The PREDIGT model, its associated coefficients for selected variables, and their imputation into the formula (P_R_ = (E + D + I) x G x T), were previously developed and described^13^. Therefore, both cohorts can be viewed as external validation datasets. A summary of the study workflow, including data preparation and performance assessment, is illustrated in Fig. 1 and detailed in the Methods section. *Model 1* permits the examination of the performance for the original version. *Model 2* highlights an approach for the implementation of the PREDIGT Score to potential population screening (Fig. 1).

### Demographics and participant characteristics

For the initial analysis, we included 245 DeNoPa subjects (PD: *n* = 135; HC: *n* = 110) and 624 PPMI individuals (PD: *n* = 428; HC: *n* = 196). The FOUND Study contained a subset of PPMI subjects (PD: *n* = 141; HC: *n* = 75). Generally, DeNoPa and PPMI participants showed comparable clinical and demographic characteristics (Table 1). Subjects in the PPMI cohort showed a higher percentage of PD patients (68.59%) than did the DeNoPa study (55.1%); in both cohorts, patients were determined to be predominantly at either Hoehn and Yahr stage-1 or −2 (Table 1). The sex ratio was similar across cohorts and disease categorization: approximately two-thirds of participants in each group were males. Median ages and ranges were comparable across cohort, sex, and disease categorization (PD, HC), although participants in the DeNoPa study were slightly older than those in PPMI (by 3 years in median age), and males were slightly older than females (Table 1); these differences were statistically insignificant (*p* > 0.05). As expected, participants in FOUND showed similar characteristics as those in PPMI.

### Performance of the original PREDIGT Score (*Model 1*)

#### PPMI + FOUND cohort

**Fig. 2:**
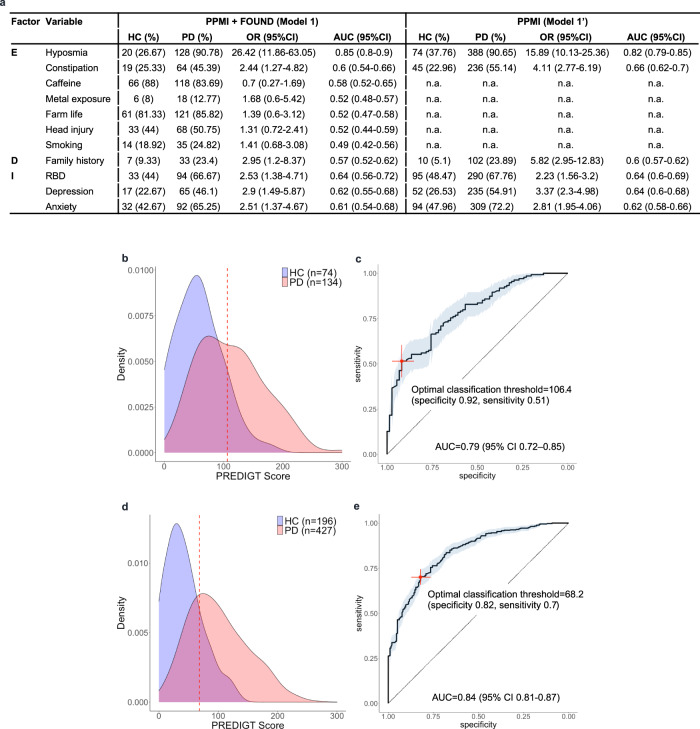
Evaluation of the original PREDIGT Score in the PPMI + FOUND cohort (Model 1; Model 1’). **a** Table of variables and their associated effect sizes, as gleaned from the OR [95% CI], the AUC values from corresponding univariate logistic regression analyses, and the number of subjects (with percentage, %) for positive cases in each group. Variables within each factor were listed in decreasing order of their AUC values; **b**, **d** score density plots of Model 1 and Model 1’, respectively; **c**, **e** ROC curves of Model 1 and Model 1’, respectively. The red, dashed lines in panels **b** and **d**, and crosses in corresponding **c** and **e** indicate the optimal threshold (score, Model 1: 106.2, Model 1’: 63.0) to distinguish the two groups by the maximum Youden Index. Light blue shading in the ROC curve indicates the bootstrap-estimated 95% CI. n.a. data not available, PPMI Parkinson’s Progression Marker Initiative, FOUND FOllow Up persons with Neurologic Disease, OR odds ratio, CI confidence interval, ROC receiver operating characteristic, AUC area under the ROC curve, RBD REM sleep behavior disorder.

Using the 11 variables listed in Fig. 2a at their previously assigned values (Supplementary Table 1) as well as those for gender and age, an individual score for each participant in the PPMI + FOUND cohort was calculated. As illustrated by the score density plot (Fig. 2b), PD patients had a higher PREDIGT Score than subjects in the HC group. The difference in mean scores (±SD) between these two groups was significant (*p* < 0.0001) at 112.8 (±55.5) and 58.6 (±39.1), respectively. The AUC value for the PREDIGT Score using the PPMI + FOUND cohort measured 0.79 (95% CI 0.72–0.85) (Fig. 2c). The original PREDIGT Score distinguished PD patients from HC subjects in PPMI with a relatively high degree of specificity at 0.92 (95% CI 0.85–0.97), but at a relatively low degree of sensitivity of 0.51 (95% CI 0.43–0.6), using 106.4 as the threshold.

*Model 1’*, which included eight variables but encompassed available data from all of the 623 participants in PPMI (PD: *n* = 427; HC: *n* = 196), was assessed next (Fig. 2a, d, e). There, the AUC value was 0.84 (95% CI 0.81–0.87), with a sensitivity of 0.7 (95% CI 0.65–0.74) and specificity of 0.82 (95% CI 0.77–0.87) using a PREDIGT Score of 68.2 as the optimal cut-off.

Several associations between included variables and PD diagnosis, such as the presence of hyposmia, constipation, a positive family history of PD, depression, anxiety, and RBD were statistically significant (Fig. 2a). Associations of most variables with increased or reduced risk of PD were congruent with the literature with the exception of smoking^18^. In PPMI + FOUND, a larger percentage of PD patients had a positive smoking history compared to controls, thus apparently contradicting the protective effect described in other studies. Hyposmia was highly informative in distinguishing between PD and HC groups within this cohort. Variables had similar effect sizes for the FOUND subset as for the PPMI cohort, as expected.

#### DeNoPa cohort

**Fig. 3:**
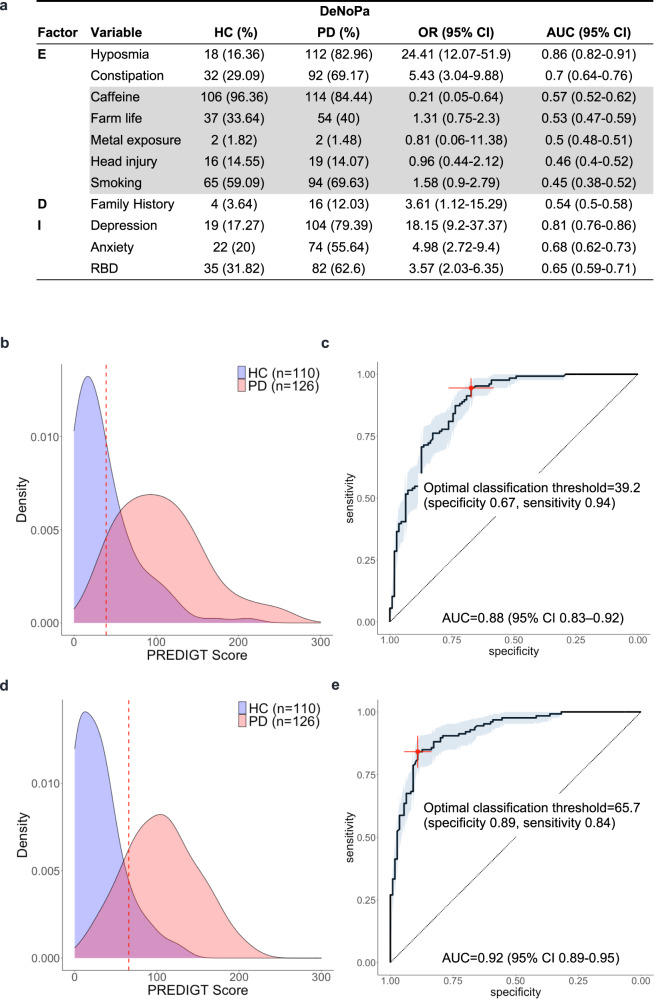
Evaluation of the original PREDIGT Score in the DeNoPa cohort (Model 1; Model 1’). **a** Table of variables and their associated effect sizes, as gleaned from the OR [95% CI], the AUC values from corresponding univariate logistic regression analyses, and the number of subjects (with percentage, %) for positive cases in each group. Variables within each factor were listed in decreasing order of their AUC values; **b**, **d** score density plots of Model 1 and Model 1’, respectively; **c**, **e**. ROC curves of Model 1 and Model 1’, respectively. The red, dashed lines in panels **b** and **d**, and crosses in corresponding **c** and **e** indicate the optimal threshold (score, Model 1: 48.1, Model 1’: 65.7) to distinguish the two groups by the maximum Youden Index. Light blue shading in the ROC curve indicates the bootstrap-estimated 95% CI. DeNoPa, De Novo Parkinson’s Study; n.a., data not available; OR odds ratio, CI confidence interval, ROC receiver operating characteristic, AUC area under the ROC curve, RBD REM sleep behavior disorder.

Using the 11 variables listed in Fig. 3a and their previously assigned values (Supplementary Table 1) as well as those for gender and age, an individual score for each participant in the DeNoPa cohort was calculated. As illustrated in the score density plot for the DeNoPa cohort (Fig. 3b), PD patients had a higher PREDIGT Score when compared with the HC group, as expected. The mean score (±SD) of the PD group was significantly higher (*p* < 0.0001) than that of healthy controls at 107.1 (±53.7*) versus* 36.6 (±38.6), respectively. The AUC value for the PREDIGT Score in the DeNoPa cohort was 0.88 (95% CI 0.83–0.92) (Fig. 3c). The PREDIGT Score distinguished PD patients from HC subjects in the DeNoPa cohort with 0.94 sensitivity (95% CI 0.9–0.98) and 0.67 specificity (95% CI 0.58–0.75), using 39.2 as the threshold.

By comparison, *Model 1’*, which excluded those environmental exposure variables that had been missing from enrollment forms in the PPMI cohort (see above, also shown in gray in Fig. 3a), was also applied to the DeNoPa cohort (Fig. 3d, e). There, the calculated AUC value was 0.92 (95% CI 0.89–0.95), with 0.84 sensitivity (95% CI 0.78–0.9) and 0.89 specificity (95% CI 0.83–0.95) using a PREDIGT Score of 65.7 as the optimum threshold.

In DeNoPa, many associations between variables from the five PREDIGT Score categories and PD, such as hyposmia, constipation, caffeinated beverage intake, family history of PD, depression, anxiety, and RBD (as diagnosed by a polysomnogram and related questionnaire), were statistically significant (Fig. 3a), as expected from the literature^13,19^. Among individual variables, the presence of hyposmia, depression, and constipation were particularly informative in distinguishing PD from HC. Of note, results for head trauma and smoking differed in the DeNoPa participants from those reported in the literature, although neither of which was statistically significant.

When the data sets from the PPMI + FOUND and DeNoPa studies were combined in the analysis, the AUCs of *Model 1* (PD, *n* = 260; HC, *n* = 184) and *Model 1’* (PD, *n* = 553; HC, *n* = 306) measured 0.84 (95% CI 0.8–0.88) and 0.87 (95% CI 0.84–0.89), respectively.

From these results, we concluded that the original PREDIGT Score discriminated newly diagnosed PD patients with relatively high accuracy from age- and sex-matched healthy individuals in the PPMI and DeNoPa cohorts. Of note, we also examined the model’s performance between male and female subjects using AUC values: within each sex, the discriminative performance of the PREDIGT Score was very similar, *i.e*., it did not perform significantly better for males or females, in the two cohorts (not shown).

### A two-step model for screening purposes (*Model 2*)

**Fig. 4:**
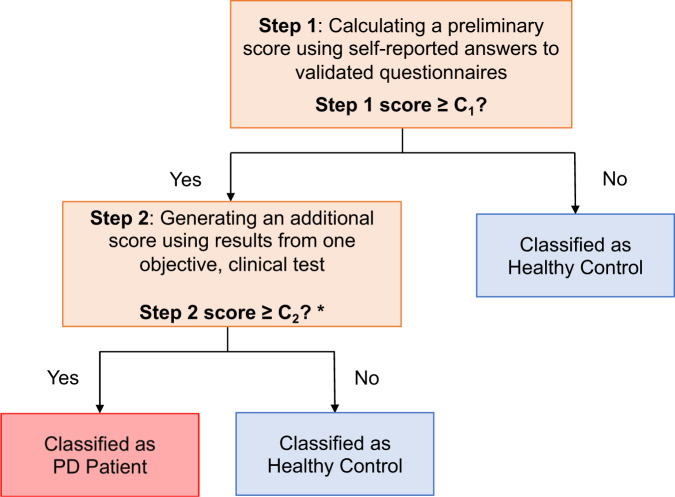
Decision tree diagram of Model 2. C_1_ and C_2_ are optimal cut-off values of Step 1 score and Step 2 score, respectively. *For select Step 2 tests (e.g., smell test), if the mean score for healthy controls (HC) is larger than that of subjects with Parkinson’s disease (PD), the decision criterion will be “Step 2 score ≤ C_2_?”.

We next explored a population screening-oriented version of the PREDIGT Score (Fig. 4), again relying on data from the DeNoPa and PPMI studies. The data encompassed subjective data from questionnaires (Supplementary Table 3) and results for seven different, objective tests (Supplementary Table 4); these had been administered at the time of each person’s baseline visit according to standardized operating procedures. We did not include results for motoric function.

#### The PREDIGT Questionnaire (Step 1 of *Model 2*)

**Fig. 5:**
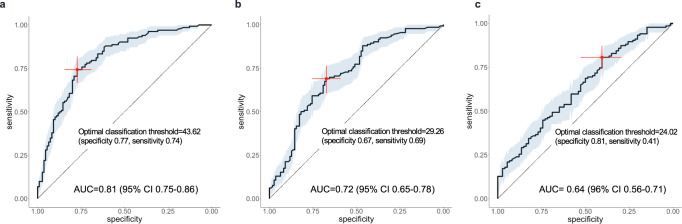
Discriminative performance of the PREDIGT Score based on self-reported questionnaires in a screening-adapted version (Model 2; Step 1). In **a**–**c** ROC curves representing the discriminative performance of: **a** a questionnaire derived from DeNoPa cohort using DeNoPa participants; **b** a questionnaire derived from the PPMI + FOUND cohort using DeNoPa participants; and **c** a questionnaire derived from PPMI + FOUND using PPMI + FOUND participants. Acronyms of cohort names as listed in Figs. 1, 2; AUC area under the ROC curve, CI confidence interval.

Self-reported answers to questions asked in clinical research forms were selected to construct cohort-specific questionnaires (see Supplementary Table 2 and Supplementary Table 3). The construct derived from the DeNoPa study (13 variables) was first applied to DeNoPa participants, while the questionnaire derived from the PPMI study (12 variables) was tested against both cohorts, i.e., PPMI + FOUND and DeNoPa (Fig. 5). The AUC value for DeNoPa subjects’ responses to the questionnaire in Step 1 was 0.81 (95% CI 0.75–0.86) in differentiating the PD group from HC subjects (Fig. 5a). With fewer questionnaires employed at the time of enrollment, the construct derived from PPMI showed a lesser performance in Step 1 of screening, as expected. When tested against DeNoPa participants, its AUC value was 0.72 (95% CI 0.65–0.78) (Fig. 5b); when tested against PPMI + FOUND participants, its AUC value was 0.64 (95% CI 0.56–0.71) (Fig. 5c). These results highlighted -among others- the importance of including self-reported information on the degree of subjects’ sense of smell, which had been captured at enrollment in the DeNoPa study but had been omitted at the time of enrollment into PPMI.

#### Determining a threshold for Step 1 in preparation for Step 2

**Fig. 6:**
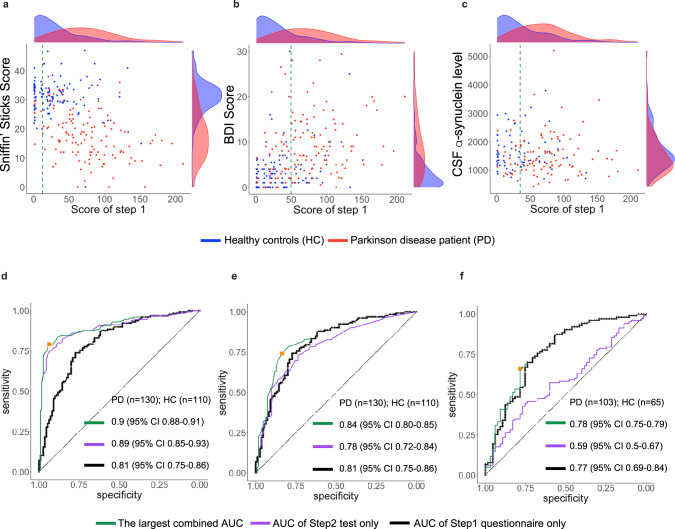
Illustration of effect sizes for steps 1 and 2 in a screening-adapted version of the PREDIGT Score (Model 2) using three distinct objective test results in Step 2. In **a** and **d** olfaction was measured by Sniffin’ Sticks test; **b** and **e** depression measured by the Beck’s Depression Inventory (BDI); **c** and **f** total α-synuclein concentration measured in the cerebrospinal fluid. Panels **a**–**c** show the relation between the Step 1 PREDIGT Questionnaire score (x-axis) and Step 2 test score (*y*-axis) by scatter plots with corresponding score densities. In **a**–**c**, blue represents the healthy control group (HC), red represents the group of Parkinson’s disease patients (PD). In **d**–**f**, corresponding, extended ROC plots are shown. Vertical, dashed, green lines in panels **a**–**c** represent the Step 1 thresholds corresponding to the largest combined AUC of Model 2, also represented in **d**–**f** by a green line. Black and purple ROC curves represent the Step 1 questionnaire and the Step 2 clinical test, separately. AUC, value for the area under the receiver operating characteristic (ROC) curve.

Determining a threshold value in Step 1 (*C*_*1*_) to achieve a high degree of discrimination between groups usually depends on the chosen study design, and in our case, also on the choice of objective marker chosen for Step 2. To aid the development of a screening-oriented variant of the PREDIGT Score (*Model 2*), we first examined three specific, clinical tests (Fig. 6) in the DeNoPa cohort, namely: the Sniffin’ Sticks performance to quantify hyposmia (a, d); the Beck’s Depression Inventory (BDI) to assess the degree of depression (b, e); and the total α-synuclein concentration in cerebrospinal fluid (CSF) (c, f). Figure 6a–c shows the variable relations between results from the Step 1 questionnaire scores (x-axis) and Step 2 objective assessment scores (y-axis) for the three selected clinical tests. Their corresponding receiver operating characteristic (ROC) curves, as shown Fig. 6d–f, compared the discriminative performances of the Step 1 questionnaire (in black), the Step 2 test by itself (in purple), and the best performing two-step model, *i.e*., largest combined AUC (in green), to classify individuals as a person with PD or as a healthy control.

#### Exploring an informative, objective test in Step 2

**Fig. 7:**
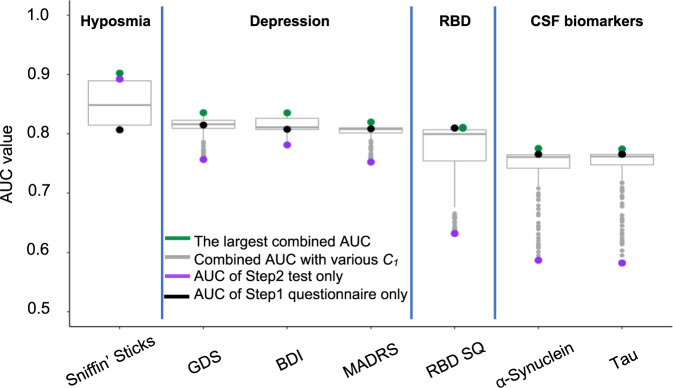
Comparison of outcomes for a screening-adapted version of the PREDIGT Score (Model 2) to separate Parkinson’s patients from healthy controls in DeNoPa. Box plots illustrate the degree of classification, as depicted by AUC values for Step 1 (PREDIGT Questionnaire score only) and Step 2 (score for one of seven objective test results) and their combination, as color-coded in the inset). The box represents the median and the two middle quartiles (25–75%). Note, for RBD SQ, the largest combined AUC value was equal to the corresponding AUC of Step 1. Therefore, we placed extra green dots on top of the corresponding boxplot. Because of missing data, especially missing CSF α-synuclein or tau levels, sample sizes in these tests were not identical. Therefore, AUC values of Step 1 (in black) were not identical for the seven tests. AUC area under the ROC curve, GDS Geriatric Depression Scale, BDI Beck’s Depression Inventory, MADRS Montgomery-Asberg Depression Scale, RBD REM sleep behavior disorder Score Sheet, CSF cerebrospinal fluid test.

In the DeNoPa cohort, the Sniffin’ Sticks test results emerged as highly discriminatory in Step 2 (Fig. 6a). Based on objectively measured olfactory function, a high degree of separation of PD patients from HC subjects could be observed both horizontally (Step 1) and vertically (Step 2). The highest combined AUC value was recorded when a cut-off of *C*_*1*_ = 12.48 was applied that advanced 70 (64%) controls and 125 (96%) PD patients into Step 2. There, *Model 2* showed an increase in AUC from 0.81 (Step 1; 95% CI 0.75–0.86) to a final AUC value of 0.9 (95% CI 0.88–0.91) when the two steps were combined. When applying a Sniffin’ Sticks test score of 25 (out of a total score of 48) as the cut-off value in Step 2, a sensitivity score of 0.79 and specificity value of 0.94 for the entire DeNoPa cohort were calculated. As shown Figs. 6d and 7, adding a smell test in Step 2 always resulted in a higher combined AUC value than relying on Step 1 alone, regardless of the selected value for *C*_*1*_.

We next tested the performance of results that addressed the presence of depression. In DeNoPa, the separation between HC subjects and PD patients by adding the BDI score, a validated tool for assessing depression, was less informative (Fig. 6b, e) than quantifying olfaction (above); this was due to a rather wide spectrum of BDI scores among PD patients. The largest combined AUC of 0.84 (95% CI 0.80–0.85) was achieved when 38 (35%) control subjects and 110 (83%) patients, each with a Step 1 PREDIGT Score of >34.88, were entered into Step 2. Using the BDI score as the Step 2 test alone, a sensitivity of 0.74 and a specificity of 0.84 were achieved.

Further, when examining the addition of a cerebrospinal fluid (CSF) marker, *e.g*., the concentration for total α-synuclein, in those subjects that were advanced to Step 2, we saw no improvement in the performance of Step 1 alone in the discrimination of PD from HC groups; rather, it reduced the combined AUC values for several of the selected C_1_ values. The latter result was attributed to the sizeable overlap in total CSF α-synuclein values between the two groups (Fig. 6c, f), which we and others previously reported^20–22^. In the subset of DeNoPa participants, who had undergone CSF testing (PD: *n* = 103; HC: *n* = 65), the largest combined AUC of 0.78 (95% CI 0.75–0.79) was achieved when 72 (70%) PD patients and 17 (26%) HCs, whose Step 1 scores were calculated as >49.5, were advanced into Step 2. The Step 2 threshold was 2.7 ng/ml in CSF α-synuclein concentration, resulting in a sensitivity of 0.66 and specificity of 0.78.

Figure 7 summarizes the comparative results for seven different candidates used in the clinical assessment of non-motor symptoms and objective biological markers in the DeNoPa population (Supplementary Table 4), which were tested in our stepwise *Model 2* of the PREDIGT Score. For each test, the corresponding box plot summarizes all the possible combined AUC values when an arbitrary *C*_*1*_ was chosen in Step 1. In the DeNoPa cohort, impaired olfaction, as tested by the Sniffin’ Sticks test, consistently generated the highest combined AUC value among all objective, non-motoric exam- and non-imaging study-based results analyzed. In addition to olfaction, the combined AUC for other clinical tests, such as for the degree of depression, as measured by three different tools, and the presence of a reduction in one of two CSF biomarkers, were also consistently higher than the AUC value calculated from Step 1 alone (Fig. 7).

Similar outcomes for the serial, two-step model were observed when testing seven different, biological variables in the PPMI + FOUND cohort (Supplementary Table 4). Although the Step 1 questionnaire derived from PPMI had not been as informative as its DeNoPa counterpart, with the main reason being that self-reported questions about participants’ sense of smell had been missing (highlighted above in Fig. 5), the combined AUC in differentiating PD from HC subjects was markedly increased when including the results of olfaction testing, such as by UPSIT in the PPMI + FOUND cohort, as Step 2 (Supplementary Figure 1). There, the AUC rose from a value of 0.64 (95% CI 0.56–0.71) to an AUC of 0.89 (95% CI 0.84–0.94) when all the PPMI + FOUND participants were entered into Step 2 and underwent olfaction assessment by UPSIT. When using an UPSIT score of 31 (out of a maximum score of 40) as the Step 2 cut-off, a sensitivity of 0.84 and specificity of 0.85 were achieved. Using other clinical test results in Step 2, such as for a previously reported polygenic risk score^5^, or the degree of depression, or the presence of anxiety or RBD, or the result of a reduction in select CSF biomarker levels (such as for total α-synuclein or total tau), a small AUC increase was observed in many -but not all- cases (Supplementary Figure 1).

We concluded from these results that in both cohorts, a serial two-step approach, which included the objective assessment of olfaction in Step 2 either by the Sniffin’ Sticks test in DeNoPa or by UPSIT in PPMI + FOUND, achieved the highest degree of accuracy to enrich for subjects with early-stage PD in a screening-type effort, as reflected by combined AUC values of 0.9 and 0.89, respectively. Similar to outcomes in the evaluation of *Model 1*, the performances of the PREDIGT Score in *Model 2* were highly comparable between male and female participants*, i.e*., we detected no sex difference.

## Discussion

Here, we evaluated the PREDIGT Score in two, well-characterized case-control cohorts. Pertaining to our objective 1, we found that the original formula (*Model 1*) and its coefficients discriminated recently diagnosed PD patients from sex- and age-matched, healthy individuals with relatively high accuracy using readily accessible data. Notably, this discrimination occurred without reliance on any results for motoric deficits that had been recorded for study participants (Figs. 2, 3).

The unexpected outcome that *Model 1’* achieved higher AUC values with fewer variables, when compared to *Model 1* (Figs. 2, 3), suggests to us that simplification of the original table of necessary variables could be explored. For example, smoking status could potentially be excluded given our results (Figs. 2a, 3a). In addition, future updates to the list of variables will integrate newly emerging epidemiological and mechanistic insights into the development of PD. There, risk elements, such as well-documented, microbial encounters, confirmed toxicant exposures, and/or other chronic conditions such as inflammatory bowel disease, hypertension, diabetes, and rosacea, will be individually evaluated for possible inclusion^23–27^. On the protective side, we will analyze cohorts that have quantified physical exercise^28^.

In the Introduction section, several published algorithms to identify patients with PD, as well as subjects in a prodromal stage, were mentioned. Compared with those models, the PREDIGT Score, which had been designed to test a hypothesis about the pathogenesis of typical PD and to use a readily accessible, simple-to-use formula, performed at similar levels. We posit that both approaches, algorithms developed based on statistical analyses as well as hypothesis-driven models, have advantages and disadvantages. Most likely, each approach will inform the other to improve performance, such as when testing revised versions in the future. Distinct classification methods may also serve different research purposes and clinical applications.

An important insight gained from our study relates to data collection in already ongoing as well as yet-to-be-planned cohort studies. Rigorously standardized research forms and applied methodologies to quantify risk modification across all potential variables, in particular within the growing fields of exposome-, genome-, and biomarker-based research activities, are necessary to better compare outcomes across cohorts. For example, DeNoPa and PPMI employed slightly different definitions of head trauma, which could explain its opposite effects on the association with PD in our univariate analysis (Figs. 2a, 3a). Further, updated research forms should include information on the possible contribution of race, ethnicity, and geography to adequately reflect differences in the worldwide incidence and prevalence rates of PD^29^.

With respect to the usage of biochemical markers, we noted that while the inclusion of CSF α-synuclein quantification did not significantly improve the performance of the PREDIGT Score in these two cohorts, future integration of results for specific variants, such as of phosphorylated α-synuclein, or for positive signals generated in templating assays in vitro, such as by RT-QuIC measurement, may evolve to be informative^30–33^. Similarly, the integration of more refined polygenic risk scores could be tested in the future^34,35^. Important from the perspective of a movement disorder, in possible revisions of the PREDIGT model both subjective as well as objective motor signs could be integrated in steps 1 and 2, respectively. These could come from different sources, *e.g*., symptoms reported by subjects, information from wearable devices, and ratings by healthcare practitioners; this, to optimize the identification of subjects at an early stage of already manifest parkinsonism or in a prodromal state with only mild bradykinesia.

The second objective of our study was to examine the PREDIGT Score’s suitability as a simple-to-use, clinical screening tool in the future for the assessment of an individual’s PD risk. To model an enrichment effort for persons at risk of PD in a wider population, or for those who may already be in a prodromal state of PD, we created a variant of the original PREDIGT Score (*Model 2*); it combines a preliminary score generated from exclusively self-reported information under Step 1 with the results of a single, objective assessment tool in Step 2 (Figs. 4–7). We demonstrate here that the combination of a questionnaire-based score and the result of an objective smell test, as carried out on those individuals with a higher preliminary score in Step 1, generated a robust performance by *Model 2* of the PREDIGT Score in distinguishing patients with PD from HC subjects in both cohorts. In practice, the Step 1 questionnaire could be helpful to the (pre-)screening of community-based populations in an inexpensive, unsupervised way to identify possible at-risk individuals for subsequent assessment in Step 2. Here, we focused on the addition of one objective score (rather than multiple tests) in Step 2 for simplicity. An added advantage could be that select tests in step 2, such as simplified smell tests^36^, could also be performed inexpensively and in an unsupervised manner outside the clinic setting. Alternatively, practitioners could choose several objective tests based on variable study designs. By extension, motor assessments (such as bradykinesia objectively measured) could also be incorporated under Step 2 of the screening-based approach and would likely increase the model’s accuracy.

In sum, we posit that employing a *Model 2-*like version of the PREDIGT Score could facilitate recruitment of neurologically still healthy persons with higher PD risk into prevention studies, at a time when such measures become available. It could also serve as a tool to screen PD subjects referred to neurologists’ offices for their initial evaluation, beginning with completion of Step 1 at home, followed by completion of Step 2, such as objective assessment of olfaction, at the time of their clinic visit. Moreover, the PREDIGT Score may also aid movement disorder specialists in their efforts to more rapidly recruit homogenous study populations, such as subjects with *versus* without hyposmia, into clinical trials for manifest PD as well as those in a prodromal state.

For the PREDIGT Score model, selection of variables and their numerical values were based on published meta-analyses previously reported by us^13^; therefore, the results reported here do not have an over-fitting bias and can be readily tested further using other cohorts and populations. The performance of the PREDIGT Score was evaluated using two well-characterized cohorts in which many (but not all) variables from our original score sheet had been captured. We found that the outcomes were comparable for a single-center study (DeNoPa) *vs*. a multi-centric (PPMI + FOUND) cohort, and for male *vs*. female participants. Accuracy of the clinical diagnosis of PD was achieved through ongoing follow-up visits in both cohorts as well as dopamine transporter (DAT) scanning of all enrolled patients in PPMI, thereby permitting robust group classification with the elimination of false positives as well as reliable algorithm assessment. The PREDIGT Questionnaire of *Model 2* (in Step 1) is based on self-reported information collected via validated questionnaires that need no involvement by a licensed, clinical practitioner. Thus, an additional, potential benefit of our PREDIGT Score model could be cost effectiveness.

The fact that the PREDIGT Score rests on a hypothesis that utilizes surrogates for several still unknown variables, such as under the category E (exposome), could be seen as a weakness of the original model. We are committed to replacing any surrogate of our current categories with the actual risk modifier in future updates, once they become available, such as from comprehensive microbiome analyses of the nasal cavity and the gastrointestinal tract in validated studies^37^.

Case-control studies encompass an inherent potential for selection bias in their recruitment. For example, due to the sex- and age-matched study design of the DeNoPa and PPMI, the effect sizes of categories ‘Gender’ and ‘Time’ (age) were not adequately represented. Similarly, details for a positive family history of PD (category D) were also limited in both cohorts’ inclusion/exclusion criteria. Consequently, the performance of the PREDIGT Score reported herein was rather conservative. We will address this limitation in future validation efforts to examine and calibrate coefficients of the PREDIGT Score in actual population settings^38^. Of note, currently ongoing studies by our team include the testing of the model’s predictive performance using prospective, longitudinal studies of neurologically healthy persons, who were enrolled after having been identified as carrying a specific risk element, *e.g*., hyposmia or REM sleep abnormalities^19,39,40^.

To further explore the operational feasibility of the PREDIGT Score model, such as in a two-tiered approach, and to probe for its accuracy to detect PD *versus* other conditions, we have recently launched a prospective study for its testing at our clinics. There, other neurological disorders, such as Alzheimer’s disease, which also frequently features hyposmia^41^, atypical parkinsonism, cases of secondary parkinsonism, and forms of dystonia as well as essential tremor, will be included. In planning it, it became apparent that the most expensive part of a PREDIGT Score-based screening approach will be the type of biological marker assessment chosen for Step 2 (invasive, yes/no; healthcare practitioner-administered, yes/no; size of cut-off chosen after Step 1, low/high; etc.).

Therefore, in our work on optimizing the PREDIGT Score and its potential transformation into practice, we are guided not only by similar efforts in the field (e.g.,^8,9^), recent successes in dementia research^42^, but foremost by five principles: (i) internal as well as external validation; (ii) simplicity in usage by lay persons and healthcare practitioners; (iii) overall ease of accessibility; (iv) relevance to current health maintenance and future interventions; and (v), last-but-not-least, cost effectiveness.

## Methods

### Source of data and participants

We used de-identified baseline data from subjects enrolled in the DeNoPa and PPMI cohorts. Of note, there was no overlap in participating individuals between the two cohorts. The former^15^ represents a single-center, observational, longitudinal study of patients with a newly established (“*de novo*”) diagnosis of PD (UK Brain Bank Criteria^43^) that were naïve to L-Dopa therapy at baseline, and of age- as well as sex-matched, neurologically healthy controls. Exclusion criteria included previously known or subsequently detected brain conditions, such as normal pressure hydrocephalus, cerebrovascular disease, features of atypical parkinsonism (*e.g*., multisystem atrophy or progressive supranuclear palsy), and medication-induced parkinsonism. Healthy controls were recruited through relatives and friends of enrolled PD subjects, other patients of the hospital-based clinic as well as through newspaper advertisements in 2009; control subjects had to be without any active, known or previously treated condition of their central nervous system. Diagnostic accuracies for study participants were ensured by ongoing follow-up visits every two years (as of 2022, 10-year follow-up visits are ongoing). Twenty-four patients classified as PD at baseline were later re-grouped as “other neurological diagnosis” (OND) and excluded from the study.

The PPMI cohort^16^ represents an international, multi-centric (33 centers), observational, longitudinal study. PD patients were diagnosed within the previous two years, confirmed with a DAT scan, and remained untreated. Individuals with other movement disorders were excluded, as described above for DeNoPa participants. Healthy controls had to have no significant neurological dysfunction, no first-degree family member of PD, and a Montreal Cognitive Assessment test score of 26–30 (out of a maximum score of 30). In PPMI, visits took place every three months during the first year and every six to 12 months up to 96 months thereafter. Environmental exposure variables, factor ‘E’ from our model, were not collected in the original PPMI study. To supplement the missing information, data from the FOUND study^17^ were used, which represents a follow-up study for a subset of PPMI participants; hence, environmental exposure data associated with PD incidence were obtained retrospectively. PPMI participants that fell into other groups (*e.g*., OND and subjects having ‘scans without evidence of a dopaminergic deficit’ (SWEDD)) were not included in the analysis.

In both cohorts, we focused on previously identified variables^13^. Data entries related to motor performances were not included in the analysis. Informed consent from all participants was obtained in DeNoPa and PPMI (FOUND) studies. Analyses of their de-identified data sets were approved by the Regulatory Ethics Board of The Ottawa Hospital (20180010-01H).

### Procedures and statistical analyses

#### Data preparation

The published, original PREDIGT Score included 30 variables associated with altered risk for developing PD^13^. Among those, 20 variables were ascertained at enrollment into the two respective cohorts. After excluding 4 variables with only free-text data entries (*e.g*., infection history) and low prevalence of exposure (*e.g*., MPTP-type neurotoxin exposure), we included 16 variables into this study. Most variables had a low rate of missing data (<5%); in such cases, we used corresponding data of the nearest follow-up visit by the same participant for data imputation. CSF biomarker data for total α-synuclein and tau proteins and genetic risk scores (GRS) were excluded from our initial analysis for two reasons: one, they had higher missing data rates (DeNoPa: 32.7%; PPMI: 12.1%); and two, univariate analysis of these three variables showed only mild to moderate association with PD diagnosis (univariate AUC 0.57–0.63). Thus, 13 variables entered phase 1 of our study (Supplementary Table 1).

#### Variable ascertainment

Environmental exposure variables and family history of PD were self-reported. Multiple instruments to evaluate non-motor symptoms were used in DeNoPa and PPMI (for screening instruments and abbreviations, see Supplementary Table 1). In DeNoPa, the presence of non-motor symptoms was self-reported using the MDS-UPDRS I, Scopa-AUT, PD NMSS, PD NMS, and PDQ-39 questionnaires. MDS-UPDRS I and Scopa-AUT were also used in PPMI for assessing non-motor aspects of PD. Questionnaires for depression (GDS, BDI, MADRS), anxiety (STAI), and REM sleep behavior (RBD SQ) were also included in at least one of the two cohorts. Sniffin’ Sticks test and UPSIT were used to quantify olfaction in DeNoPa and PPMI, respectively. We classified patients as having normosmia, hyposmia, or anosmia by using published cut-off values^44,45^. Although PPMI participants’ overall genetic risk of having PD were quantified using a previously established, genetic risk score^5^, detailed genetic risk of PD was not readily available from most of the patients and family members in the clinic; hence, self-reported family history of PD were used as a surrogate for genetic risk information.

#### Univariate analyses of risk modulators

Although coefficient assignment for variables was not derived from PPMI and/or DeNoPa (but previously established^13^), univariate analysis in each model was used to confirm their association with the diagnosis of PD, such as via: the number and percentage of positive cases in each group; the odds ratio (OR); and an univariate AUC.

#### Constructing the PREDIGT questionnaire

For non-motor aspects of PD (*i.e*., constipation, hyposmia, depression, anxiety, and RBD), the PREDIGT Questionnaire was directly informed by the five questionnaires (MDS-UPDRS I; Scopa-AUT; PD NMSS; PD NMS; and PDQ-39) included in the DeNoPa clinical research forms. In PPMI, clinical research forms entailed two related questionnaires (MDS-UPDRS I; Scopa-AUT) but none related to olfaction. Thus, additional analyses with fewer shared variables were performed using both PPMI + FOUND and DeNoPa cohorts. The list of questionnaires analyzed are shown in Supplementary Table 2; those selected for model purposes are listed in Supplementary Table 3.

#### Calculating an individual’s PREDIGT Score

After reviewing available information in the two cohorts, 13 variables entered phase 1 of our study (Supplementary Table 1). These were transformed into a binary format (positive/negative) or ordinal format with multiple levels and then multiplied by the previously published coefficients, based on criteria shown in Supplementary Table 1^13^. The PREDIGT Score of each participant was then calculated as per the formula: P_R_ = (E + D + I) x G x T. In order to ensure all participants had scores >0 (that would increase with progression in age), age-associated minimal values for factors ‘E’, ‘D’, and ‘I’ were included, as previously described^13^ (see also details in Supplementary Table 1).

#### Model performance

We evaluated the discrimination ability of the PREDIGT Score between PD patients and HC in a cross-sectional analysis. ROC curves and AUC values were calculated. Confidence intervals (CI) of reported AUC values were estimated using bootstrapping with 1000 replications. Reported sensitivity and specificity were relative to the optimal threshold identified using the Youden Index^46^. Score density plots were used to illustrate the degree of distinction between groups. Two versions of the PREDIGT Score were assessed (*Model 1* and *Model 2*).

##### Model 1: PREDIGT Score

The original formula was tested using DeNoPa and PPMI + FOUND data separately to generate a PREDIGT Score of each participant (Supplementary Table 1, Fig. 1).

##### Model 2: 2-step PREDIGT Score

For population screening purposes, we created a two-step model using first self-reported variables (Step 1) and a single, objective test (Step 2). The screening-focused questionnaire was derived from the original PREDIGT Score model (Supplementary Table 3) and was assembled using exclusively self-reported data. The screening-oriented PREDIGT Score was then calculated using the same formula and coefficients as above. The Step 1 threshold, *C*_*1*_, was determined by the largest combined AUC that could be achieved. Objective markers assessed in Step 2 are listed in Supplementary Table 4; the Step 2 threshold, *C*_*2*_, was determined by the maximum of Youden Index.

Statistical analyses were performed using ‘R’ (version 3.6.0). Library ‘rms’^47^ was used for logistic regression; all plots were generated using ‘ggplot2’^48^ in R.

## Supplementary information

Supplementary Figure and Tables

## Data Availability

Applications for DeNoPa data are reviewed by the study investigators (Drs. Brit Mollenhauer and Claudia Trenkwalder; see also at https://www.denopa.de/Home.29.0.html). Applications for PPMI (FOUND) data are reviewed by the PPMI steering committee (https://www.ppmi-info.org/access-data-specimens/download-data).

## Code availability

All analyses were performed using R version 3.6.0. Codes for data analysis is available via https://github.com/JuanLiOHRI/PREDIGT.
