# Supplementary material for: Evaluation of the PREDIGT score’s performance in identifying newly diagnosed Parkinson’s patients without motor examination
Source: NPJ Parkinsons Dis. 2022 Jul 29;8:94. doi: 10.1038/s41531-022-00360-5 (PMC9338052; doi:10.1038/s41531-022-00360-5)
Supplement: Supplementary file 1 — Supplementary Figure and Tables [file 41531_2022_360_MOESM1_ESM.pdf]

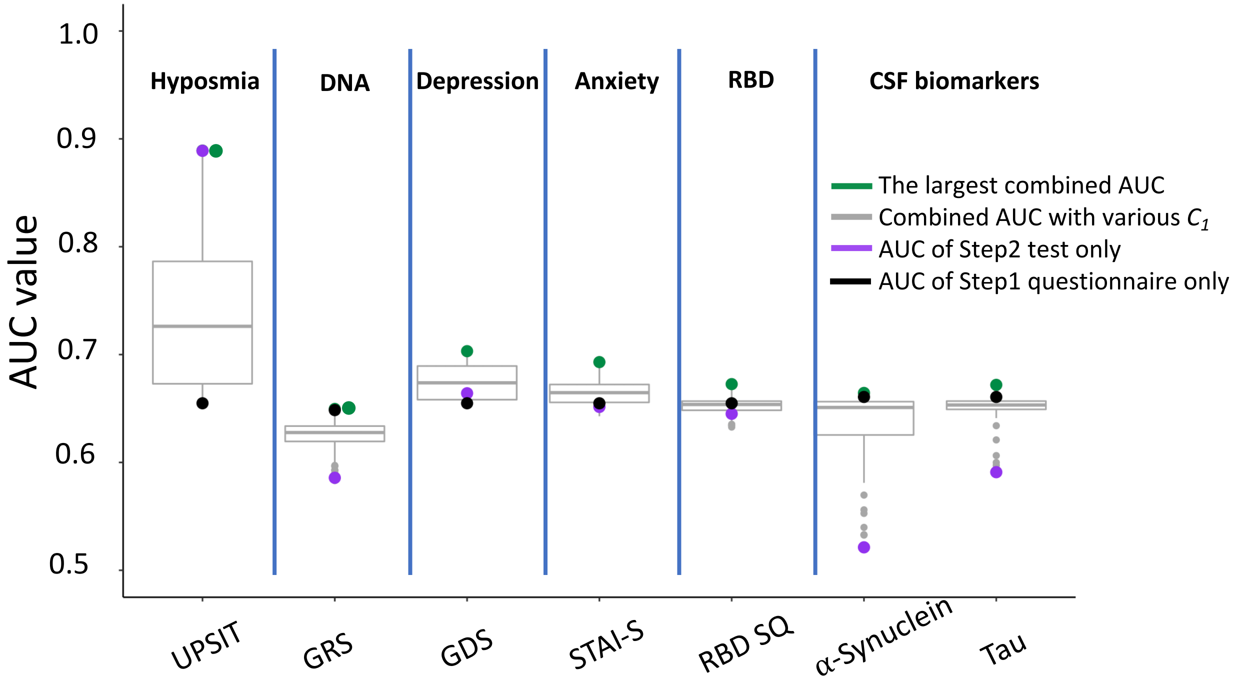

Supplementary Figure 1: Comparison of outcomes for a screening-adapted version of the PREDIGT Score (*Model 2*) to separate Parkinson's patients from healthy controls in PPMI. Box plots illustrate the degree of classification, as depicted by AUC values for Step 1 (PREDIGT Questionnaire score only) and Step 2 (score for one of seven objective test results) and their combination, as color-coded in the inset). The box represents the median and the two middle quartiles (25–75%). Note, for GRS, the largest combined AUC value was equal to the corresponding AUCs of Step 1; and the largest combined AUC value when using UPSIT was the same as the corresponding AUC for the UPSIT test itself. Therefore, we placed extra green dots on top of the corresponding boxplots. Because of missing data, sample sizes in these tests were not identical. Therefore, AUC values of Step 1 (in black) were not identical. AUC, area under the ROC curve; UPSIT, University of Pennsylvania Smell Identification Test; GRS, genetic risk score; GDS, Geriatric Depression Scale; STAI-S, State-Trait Anxiety Inventory-State score; RBD, REM sleep behavior disorder Score Sheet; CSF, cerebrospinal fluid test.

SUPPLEMENTARY TABLES

Supplementary Table 1:

Select variables and their previously assigned values within five categories that are entered into the PREDIGT Score formula [P_R=(E+D+I)xGxT)].

Factor	Assigned value	DeNoPa	PPMI	FOUND
**Factor E: Exposome**				
Metal exposure	0.5	Self-reported	n.a.	Self-reported
Pesticide exposure/Farm life	0.25	Self-reported	n.a.	Self-reported
Head trauma<br>Concussive event(s)<br>Subconcussive event(s)	 1<br>0.5	Self-reported	MH	Self-reported
Constipation	0.5	MDS UPDRS: 1.11<br>SCOPA AUT: 05, 06<br>PD NMS: 05<br>PD NMSS: 21	MDS UPDRS: 1.11<br>SCOPA AUT: 05, 06	PPMI
Reduced olfaction<br>Anosmia<br>Hyposmia	 1<br>0.5	PD NMS 02<br>PD NMSS 28<br>**Sniffin' Sticks 16**	**UPSIT**	PPMI
Smoking<br>Current >= 20 years<br>Current 11-19 years<br>Past >= 20 years<br>Past 11-19 years<br>Any <= 10 years	 -0.75<br>-0.5<br>-0.25<br>-0.125<br>-0.0625	Self-reported	n.a.	Self-reported
Caffeine intake<br>>= 2 cups/day recent<br>>= 1 cups/day recent	 -0.25<br>-0.125	Self-reported	n.a.	Self-reported
Minimal risk score of 'E'<br>Age of proband<br><= 50 years<br>51-59 years<br>60-69 years<br>70-79 years<br>>= 80 years	 <br>0<br>0.005<br>0.0075<br>0.02<br>0.03			
**Factor D: DNA**				
Family history of disease<br>1st degree relative<br>2nd degree relative<br>3rd degree relative	 0.5<br>0.25<br>0.125	Self-reported	Self-reported	PPMI

Minimal risk score of ‘D’	0.01			
**Factor I: Initiation of tissue response**				
Presence of depression	0.25	MDS UPDRS: 1.3<br>PD NMS: 16<br>PD NMSS: 10<br>UPDRS 1: 3<br>PDQ39: 17<br>**GDS; BDI; MADRS**	MDS UPDRS: 1.3<br>**GDS**	PPMI
Presence of anxiety	0.25	MDS UPDRS: 1.4<br>PD NMS: 17<br>PD NMSS: 09<br>PDQ39: 21	MDS UPDRS: 1.4<br>**STAI**	PPMI
Presence of RBD	0.25	PD NMS: 25<br>**RBD SQ**	**RBD SQ**	PPMI
Minimal risk score of ‘I’<br>Age of proband<br><= 50 years<br>51-59 years<br>60-69 years<br>70-79 years<br>>= 80 years	 <br>0<br>0.001<br>0.002<br>0.003<br>0.004			
**Factor G: Gender (Sex)**<br>Male<br>Female	 1.2<br>0.8	Sex	Sex	PPMI
**Factor T: Time (Age)**<br>Age	Subject’s age	Age at baseline visit	Age at baseline visit	PPMI

Data collection in DeNoPa and PPMI+FOUND cohorts: most variables were collected using self-reported questions. Additional instruments/assessments were highlighted in bold. DeNoPa = *De Novo* Parkinson Study. PPMI = Parkinson’s Progression Marker Initiative. FOUND = Follow Up persons with Neurologic Disease Study. PD = Parkinson’s disease. MH = Medical History. MDS-UPDRS = Movement Disorder Society-Sponsored Revision of the Unified Parkinson’s Disease Rating Scale. Scopa-AUT = Scale for Outcomes in PD for Autonomic Symptoms. PD NMS = Non-Movement Problems in Parkinson’s. PD NMSS = Non-Motor Symptom assessment scale for Parkinson’s Disease. UPSIT = University of Pennsylvania Smell Identification Test. UPDRS = Unified Parkinson’s Disease Rating Scale. GDS = Geriatric Depression Scale (Short Version). BDI = Beck's Depression Inventory. MADRS = Montgomery-Asberg Depression

Scale. STAI = State Trait Anxiety Inventory. RBD = REM Sleep Behavior Disorder. RBD SQ = The REM Sleep Behavior Disorder Screening Questionnaire.

Supplementary Table 2:

Questions used in DeNoPa and PPMI regarding five non-motor symptoms entered in the PREDIGT model.

Variable	Question	DeNoPa		PPMI	
		OR (95% CI)	AUC (95% CI)	OR (95% CI)	AUC (95% CI)
**Constipation**	**SCOPA.AUT.06**	**4.95 (2.79-8.95)**	**0.7 (0.64-0.76)**	**4.14 (2.76-6.31)**	**0.66 (0.63-0.7)**
	SCOPA.AUT.05	4.75 (2.12-11.75)	0.61 (0.56-0.65)	3.53 (2.16-5.99)	0.61 (0.57-0.64)
	PD.NMS.05	6.48 (2.38-22.15)	0.6 (0.55-0.64)		
	MDS.UPDRS.1.11	5.96 (2.18-20.45)	0.59 (0.55-0.63)	3.51 (2.17-5.9)	0.61 (0.57-0.64)
	PD.NMSS.21.Severity	5.46 (1.78-22.44)	0.57 (0.53-0.6)		
	PD.NMSS.21.Frequency	5.46 (1.78-22.44)	0.57 (0.53-0.6)		
**Hyposmia**	**PD.NMS.02**	**14.82 (6.94-34.78)**	**0.75 (0.71-0.8)**		
	PD.NMSS.28.Severity	11.87 (5.56-27.83)	0.73 (0.68-0.78)		
	PD.NMSS.28.Frequency	14.23 (6.29-36.6)	0.74 (0.69-0.79)		
**Depression**	**PDQ39.17**	**5.38 (3-9.86)**	**0.71 (0.65-0.77)**		
	MDS.UPDRS.1.03	16.14 (6.13-54)	0.69 (0.65-0.74)	**2.24 (1.36-3.8)**	**0.56 (0.53-0.59)**
	PD.NMSS.10.Severity	8.69 (3.67-23.9)	0.66 (0.61-0.7)		
	PD.NMSS.10.Frequency	8.69 (3.67-23.9)	0.66 (0.61-0.7)		
	PD.NMS.16	4.35 (2.28-8.65)	0.64 (0.59-0.7)		
**Anxiety**	**PDQ39.21**	**4.48 (2.45-8.43)**	**0.68 (0.62-0.74)**		
	MDS.UPDRS.1.04	8.85 (2.61-46.91)	0.59 (0.55-0.62)	**2.31 (1.52-3.57)**	**0.58 (0.55-0.62)**
	PD.NMSS.09.Severity	4.35 (1.67-13.42)	0.58 (0.54-0.62)		
	PD.NMSS.09.Frequency	4.35 (1.67-13.42)	0.58 (0.54-0.62)		
	PD.NMS.17	4.44 (1.21-24.55)	0.54 (0.51-0.57)		
**RBD**	**PD.NMS.25**	**3.35 (1.64-7.27)**	**0.6 (0.55-0.65)**		
	RBD.SQ.06 sum 1-2	1.95 (1.09-3.53)	0.58 (0.52-0.64)	**1.68 (1.16-2.43)**	**0.57 (0.53-0.61)**
	RBD.SQ.06 sum 1-4	1.75 (0.99-3.12)	0.57 (0.51-0.63)	1.76 (1.22-2.55)	0.57 (0.53-0.62)

For each variable, the questions were ordered by their AUC values in the DeNoPa cohort. The most informative questions were also highlighted by bold.

Supplementary Table 3:

Questions included into Step 1 of *Model 2* questionnaire.

Factor	Variable	Questionnaire derived from DeNoPa	Questionnaire derived from PPMI
**E**	**Metal exposure**	**Metal exposure?**<br>0 = No<br>1 = Yes	**Metal exposure?**<br>0 = No<br>1 = Yes
	**Pesticide exposure/ Farm life**	**Pesticide/herbicide and/or farm life?**<br>0 = No<br>1 = Yes	**Pesticide/herbicide and/or farm life?**<br>0 = No<br>1 = Yes
	**Head trauma**	**Severe Head Trauma?**<br>0 = No<br>1 = Yes, didn't lose consciousness<br>2 = Yes, lost consciousness	**Severe Head Trauma?**<br>0 = No<br>1 = Yes, didn't lose consciousness<br>2 = Yes, lost consciousness
	**Hyposmia**	**PD.NMS.02**<br>**Have you experienced any of the following in the last month?**<br>**Loss or change in your ability to taste or smell.**<br>0 = No<br>1 = Yes	n.a
	**Constipation**	**SCOPA.AUT.06**<br>**In the past month, did you have to strain hard to pass stools?**<br>0 = never<br>1 = sometimes<br>2 = regularly<br>3 = often	**SCOPA.AUT.06**<br>**In the past month, did you have to strain hard to pass stools?**<br>0 = never<br>1 = sometimes<br>2 = regularly<br>3 = often
	**Smoking**	**Smoking history:**<br>0 = No<br>1 = Any <= 10 years<br>2 = Past 11-19 years<br>3 = Past >= 20 years<br>4 = Current 11-19 years<br>5 = Current >= 20 years	**Smoking history:**<br>0 = No<br>1 = Any <= 10 years<br>2 = Past 11-19 years<br>3 = Past >= 20 years<br>4 = Current 11-19 years<br>5 = Current >= 20 years
	**Caffeine intake**	**Caffeinated beverage intake:**<br>0 = No<br>1 = over 1 cups/day recent<br>2 = over 2 cups/day recent	**Caffeinated beverage intake:**<br>0 = No<br>1 = over 1 cups/day recent<br>2 = over 2 cups/day recent
**D**	**Family history**	**Family history of PD:**<br>1 = 1st degree relative<br>2 = 2nd degree relative<br>3 = 3rd degree relative	**Family history of PD:**<br>1 = 1st degree relative<br>2 = 2nd degree relative<br>3 = 3rd degree relative

**I**	**Depression**	**PDQ39.17**<br>**Due to having Parkinson's disease, how often during the last month have you felt depressed?**<br>0 = never<br>1 = Occasionally<br>2 = Sometimes<br>3 = Often<br>4 = Always (or cannot do at all)	**MDS.UPDRS.1.03**<br>**DEPRESSED MOOD**<br>**Over the past week have you felt low, sad, hopeless, or unable to enjoy things? If yes, was this feeling for longer than one day at a time? Did it make it difficult for you carry out your usual activities or to be with people?**<br>0: Normal: No depressed mood.<br>1: Slight: Episodes of depressed mood that are not sustained for more than one day at a time. No interference with patient's ability to carry out normal activities and social interactions.<br>2: Mild: Depressed mood that is sustained over days, but without interference with normal activities and social interactions.<br>3: Moderate: Depressed mood that interferes with, but does not preclude the patient's ability to carry out normal activities and social interactions.<br>4: Severe: Depressed mood precludes patient's ability to carry out normal activities and social interactions.
	**Anxiety**	**PDQ39.21**<br>**Due to having Parkinson's disease, how often during the last month have you felt anxious?**<br>0 = never<br>1 = Occasionally<br>2 = Sometimes<br>3 = Often<br>4 = Always (or cannot do at all)	**MDS.UPDRS.1.04**<br>**ANXIOUS MOOD**<br>**Over the past week have you felt nervous, worried, or tense? If yes, was this feeling for longer than one day at a time? Did it make it difficult for you to follow your usual activities or to be with other people?**<br>0: Normal: No anxious feelings.<br>1: Slight: Anxious feelings present but not sustained for more than one day at a time. No interference with patient's ability to carry out normal activities and social interactions.<br>2: Mild: Anxious feelings are sustained over more than one day at a time, but without interference with patient's ability to carry out normal activities and social interactions.<br>3: Moderate: Anxious feelings interfere with, but do not preclude, the patient's ability to carry out normal activities and social interactions.<br>4: Severe: Anxious feelings preclude patient's ability to carry out normal activities and social interactions.
	**RBD**	**PD.NMS.25**<br>**Have you experienced any of the following in the last month?**<br>**Talking or moving about in your sleep as if you are 'acting' out a dream.**<br>0 = No<br>1 = Yes	**RBD.SQ.6.1 and 6.2**<br>**I have or had the following phenomena during my dreams:**<br>**6.1. speaking, shouting, swearing, laughing loudly**<br>**6.2. sudden limb movements, "fights"**<br>0 = No<br>1 = Yes
**G**	**Sex**	**Biological sex:**<br>1 = Female<br>2 = Male	**Biological sex:**<br>1 = Female<br>2 = Male
**T**	**Age**	**Participant's age at baseline visit.**	**Participant's age at baseline visit.**

Supplementary Table 4:

Candidate objective tests for Step 2 in *Model 2*.

Factor	Variable	Objective tests used in DeNoPa	Objective tests used in PPMI
E	Hyposmia	Sniffin’ Sticks	University of Pennsylvania Smell Identification Test (UPSIT)
D	DNA	n.a.	Genetic Risk Score (GRS)
I	Depression	Geriatric Depression Scale (GDS);<br>Beck's Depression Inventory (BDI);<br>Montgomery-Asberg Depression Scale (MADRS)	Geriatric Depression Scale (GDS)
	Anxiety	n.a.	State Trait Anxiety Inventory State (STAI-S)
	RBD	The REM Sleep Behavior Disorder Screening Questionnaire (RBD SQ)	The REM Sleep Behavior Disorder Screening Questionnaire (RBD SQ)
	CSF biomarkers	α-synuclein<br>Tau	α-synuclein<br>Tau
